# Supplementary material for: A Statistical Analysis of Response and Recovery Times: The Case of Ethanol Chemiresistors Based on Pure SnO2
Source: Sensors (Basel). 2022 Aug 23;22(17):6346. doi: 10.3390/s22176346 (PMC9459747; doi:10.3390/s22176346)
Supplement: Supplementary file 1 [file sensors-22-06346-s001.zip › sensors-1851110-supplementary.pdf]

Supplementary material of

A Statistical Analysis of Response and Recovery Times: The Case of Ethanol Chemiresistors Based on Pure SnO2

Andrea Ponzoni[1,2]
[1] National Institute of Optics (INO) Unit of Brescia, National Research Council (CNR), 25123 Brescia, Italy; andrea.ponzoni@ino.cnr.it; Tel.: +39-030-3711440
[2] National Institute of Optics (INO) Unit of Lecco, National Research Council (CNR), 23900 Lecco, Italy

Table S1. List of articles used as source for analysis

N.	Article
1	Cho, Y.H.; Liang, X.; Kang, Y.C.; Lee, J.-H. Ultrasensitive Detection of Trimethylamine Using Rh-Doped SnO2 Hollow Spheres Prepared by Ultrasonic Spray Pyrolysis. *Sens. Actuators B Chem.* **2015**, *207*, 330–337, doi:10.1016/j.snb.2014.10.001.
2	Choi, K.S.; Park, S.; Chang, S.-P. Enhanced Ethanol Sensing Properties Based on SnO2 Nanowires Coated with Fe2O3 Nanoparticles. *Sens. Actuators B Chem.* **2017**, *238*, 871–879, doi:10.1016/j.snb.2016.07.146.
3	Cui, Y.; Zhang, M.; Li, X.; Wang, B.; Wang, R. Investigation on Synthesis and Excellent Gas-Sensing Properties of Hierarchical Au-Loaded SnO2 Nanoflowers. *J. Mater. Res.* **2019**, *34*, 2944–2954, doi:10.1557/jmr.2019.249.
4	Fan, X.-X.; He, X.-L.; Li, J.-P.; Gao, X.-G.; Jia, J. Ethanol Sensing Properties of Hierarchical SnO2 Fibers Fabricated with Electrospun Polyvinylpyrrolidone Template. *Vacuum* **2016**, *128*, 112–117, doi:10.1016/j.vacuum.2016.03.016.
5	Francioso, L.; De Pascali, C.; Creti, P.; Radogna, A.V.; Capone, S.; Taurino, A.; Epifani, M.; Baldacchini, C.; Bizzarri, A.R.; Siciliano, P.A. Nanogap Sensors Decorated with SnO2 Nanoparticles Enable Low-Temperature Detection of Volatile Organic Compounds. *ACS Appl. Nano Mater.* **2020**, *3*, 3337–3346, doi:10.1021/acsanm.0c00066.
6	Gu, C.D.; Zheng, H.; Wang, X.L.; Tu, J.P. Superior Ethanol-Sensing Behavior Based on SnO2 Mesocrystals Incorporating Orthorhombic and Tetragonal Phases. *RSC Adv.* **2015**, *5*, 9143–9153, doi:10.1039/C4RA13940B.
7	Gulevich, D.; Rumyantseva, M.; Gerasimov, E.; Khmelevsky, N.; Tsvetkova, E.; Gaskov, A. Synergy Effect of Au and SiO2 Modification on SnO2 Sensor Properties in VOCs Detection in Humid Air. *Nanomaterials* **2020**, *10*, 813, doi:10.3390/nano10040813.
8	Guo, J.; Zhang, J.; Gong, H.; Ju, D.; Cao, B. Au Nanoparticle-Functionalized 3D SnO2 Microstructures for High Performance Gas Sensor. *Sens. Actuators B Chem.* **2016**, *226*, 266–272, doi:10.1016/j.snb.2015.11.140.
9	Guo, W.; Wang, Z. Composite of ZnO Spheres and Functionalized SnO2 Nanofibers with an Enhanced Ethanol Gas Sensing Properties. *Mater. Lett.* **2016**, *169*, 246–249, doi:10.1016/j.matlet.2016.01.118.
10	Hoa, L.T.; Cuong, N.D.; Hoa, T.T.; Khieu, D.Q.; Long, H.T.; Quang, D.T.; Hoa, N.D.; Hieu, N.V. Synthesis, Characterization, and Comparative Gas Sensing Properties

	of Tin Dioxide Nanoflowers and Porous Nanospheres. *Ceram. Int.* **2015**, *41*, 14819–14825, doi:10.1016/j.ceramint.2015.08.003.
11	Hyun, S.K.; Sun, G.-J.; Lee, J.K.; Lee, C.; In Lee, W.; Kim, H.W. Ethanol Gas Sensing Using a Networked PbO-Decorated SnO2 Nanowires. *Thin Solid Films* **2017**, *637*, 21–26, doi:10.1016/j.tsf.2017.02.054.
12	Jayababu, N.; Poloju, M.; Shruthi, J.; Reddy, M.V.R. Semi Shield Driven P-n Heterostructures and Their Role in Enhancing the Room Temperature Ethanol Gas Sensing Performance of NiO/SnO2 Nanocomposites. *Ceram. Int.* **2019**, *45*, 15134–15142, doi:10.1016/j.ceramint.2019.04.255.
13	Jiang, X.H.; Ma, S.Y.; Sun, A.M.; Zhang, Z.M.; Jin, W.X.; Wang, T.T.; Li, W.Q.; Xu, X.L.; Luo, J.; Cheng, L.; et al. Hydrothermal Self-Assembly of Novel Porous Flower-like SnO2 Architecture and Its Application in Ethanol Sensor. *Appl. Surf. Sci.* **2015**, *355*, 1192–1200, doi:10.1016/j.apsusc.2015.08.014.
14	Jiang, Z.; Jiang, T.; Wang, J.; Wang, Z.; Xu, X.; Wang, Z.; Zhao, R.; Li, Z.; Wang, C. Ethanol Chemiresistor with Enhanced Discriminative Ability from Acetone Based on Sr-Doped SnO2 Nanofibers. *J. Colloid Interface Sci.* **2015**, *437*, 252–258, doi:10.1016/j.jcis.2014.09.056.
15	Kim, B.-Y.; Cho, J.S.; Yoon, J.-W.; Na, C.W.; Lee, C.-S.; Ahn, J.H.; Kang, Y.C.; Lee, J.-H. Extremely Sensitive Ethanol Sensor Using Pt-Doped SnO2 Hollow Nanospheres Prepared by Kirkendall Diffusion. *Sens. Actuators B Chem.* **2016**, *234*, 353–360, doi:10.1016/j.snb.2016.05.002.
16	Kotchasak, N.; Wisitsoraat, A.; Tuantranont, A.; Phanichphant, S.; Yordsri, V.; Liewhiran, C. Highly Sensitive and Selective Detection of Ethanol Vapor Using Flame-Spray-Made CeOx-Doped SnO2 Nanoparticulate Thick Films. *Sens. Actuators B Chem.* **2018**, *255*, 8–21, doi:10.1016/j.snb.2017.08.027.
17	Kuang, X.; Liu, T.; Li, T.; Zeng, W.; Peng, X.; Zhang, H. Hydrothermal Synthesis of SnO2 Hierarchical Nanostructures and Their Gas Sensing Properties. *Mater. Technol.* **2016**, *31*, 260–265, doi:10.1179/1753555715Y.0000000051.
18	Kuang, X.; Liu, T.; Shi, D.; Wang, W.; Yang, M.; Hussain, S.; Peng, X.; Pan, F. Hydrothermal Synthesis of Hierarchical SnO2 Nanostructures Made of Superfine Nanorods for Smart Gas Sensor. *Appl. Surf. Sci.* **2016**, *364*, 371–377, doi:10.1016/j.apsusc.2015.12.172.
19	Lee, D.H.; Kang, S.K.; Pak, Y.; Lim, N.; Lee, R.; Kumaresan, Y.; Lee, S.; Lee, C.; Ham, M.-H.; Jung, G.Y. Transfer of Preheat-Treated SnO2 via a Sacrificial Bridge-Type ZnO Layer for Ethanol Gas Sensor. *Sens. Actuators B Chem.* **2018**, *255*, 70–77, doi:10.1016/j.snb.2017.08.025.
20	Lee, S.-H.; Galstyan, V.; Ponzoni, A.; Gonzalo-Juan, I.; Riedel, R.; Dourges, M.-A.; Nicolas, Y.; Toupance, T. Finely Tuned SnO2 Nanoparticles for Efficient Detection of Reducing and Oxidizing Gases: The Influence of Alkali Metal Cation on Gas-Sensing Properties. *ACS Appl. Mater. Interfaces* **2018**, *10*, 10173–10184, doi:10.1021/acsami.7b18140.
21	Li, F.; Gao, X.; Wang, R.; Zhang, T. Design of WO3-SnO2 Core-Shell Nanofibers and Their Enhanced Gas Sensing Performance Based on Different Work Function. *Appl. Surf. Sci.* **2018**, *442*, 30–37, doi:10.1016/j.apsusc.2018.02.122.
22	Li, H.; Zhu, D.; Yang, Z.; Lu, W.; Pu, Y. The Ethanol-Sensitive Property of Hierarchical MoO3-Mixed SnO2 Aerogels via Facile Ambient Pressure Drying. *Appl. Surf. Sci.* **2019**, *489*, 384–391, doi:10.1016/j.apsusc.2019.05.368.

23	Li, H.; Chu, S.; Ma, Q.; Li, H.; Che, Q.; Wang, J.; Wang, G.; Yang, P. Multilevel Effective Heterojunctions Based on SnO2/ZnO 1D Fibrous Hierarchical Structure with Unique Interface Electronic Effects. *ACS Appl. Mater. Interfaces* **2019**, *11*, 31551–31561, doi:10.1021/acsami.9b10410.
24	Li, R.; Chen, S.; Lou, Z.; Li, L.; Huang, T.; Song, Y.; Chen, D.; Shen, G. Fabrication of Porous SnO2 Nanowires Gas Sensors with Enhanced Sensitivity. *Sens. Actuators B Chem.* **2017**, *252*, 79–85, doi:10.1016/j.snb.2017.05.161.
25	Li, S.-H.; Meng, F.-F.; Chu, Z.; Luo, T.; Peng, F.-M.; Jin, Z. Mesoporous SnO2 Nanowires: Synthesis and Ethanol Sensing Properties. *Adv. Condens. Matter Phys.* **2017**, *2017*, 9720973, doi:10.1155/2017/9720973.
26	Li, T.; Zeng, W. New Insight into the Gas Sensing Performance of SnO2 Nanorod-Assembled Urchins Based on Their Assembly Density. *Ceram. Int.* **2017**, *43*, 728–735, doi:10.1016/j.ceramint.2016.10.001.
27	Li, T.; Zeng, W.; Zhao, W. Gas Sensing Performance of Multiple SnO2 1D Nanostructures Based on Their Interconnect Manner. *Mater. Lett.* **2016**, *167*, 230–233, doi:10.1016/j.matlet.2016.01.013.
28	Lian, X.; Li, Y.; Zhu, J.; Zou, Y.; Liu, X.; An, D.; Wang, Q. Synthesis of Coryphantha Elephantidens-like SnO2 Nanospheres and Their Gas Sensing Properties. *Curr. Appl. Phys.* **2019**, *19*, 849–855, doi:10.1016/j.cap.2019.04.016.
29	Liang, Y.-C.; Lee, C.-M.; Lo, Y.-J. Reducing Gas-Sensing Performance of Ce-Doped SnO2 Thin Films through a Cosputtering Method. *RSC Adv.* **2017**, *7*, 4724–4734, doi:10.1039/C6RA25853K.
30	Ling-min, Y.; Sheng, L.; Bing, Y.; Miao-miao, H.; Meng-di, K.; Xinhui, F. A Highly Sensitive Ethanol Gas Sensor Based on Mesoporous SnO2 Fabricated from a Facile Double-Surfactant Template Method. *Mater. Lett.* **2015**, *158*, 409–412, doi:10.1016/j.matlet.2015.06.047.
31	Liu, S.; Zhang, Y.; Yu, B.; Wang, Z.; Zhao, H.; Zhou, N.; Zhang, T. Solvent-Free Infiltration Method to Prepare Mesoporous SnO2 Templated by SiO2 Nanoparticles for Ethanol Sensing. *Sens. Actuators B Chem.* **2015**, *210*, 700–705, doi:10.1016/j.snb.2015.01.037.
32	Liu, Y.; Jiao, Y.; Zhang, Z.; Qu, F.; Umar, A.; Wu, X. Hierarchical SnO2 Nanostructures Made of Intermingled Ultrathin Nanosheets for Environmental Remediation, Smart Gas Sensor, and Supercapacitor Applications. *ACS Appl. Mater. Interfaces* **2014**, *6*, 2174–2184, doi:10.1021/am405301v.
33	Liu, Y.; Li, X.; Wang, Y.; Li, X.; Cheng, P.; Zhao, Y.; Dang, F.; Zhang, Y. Hydrothermal Synthesis of Au@SnO2 Hierarchical Hollow Microspheres for Ethanol Detection. *Sens. Actuators B Chem.* **2020**, *319*, 128299, doi:10.1016/j.snb.2020.128299.
34	Liu, Y.; Yang, P.; Li, J.; Matras-Postolek, K.; Yue, Y.; Huang, B. Morphology Adjustment of SnO2 and SnO2/CeO2 One Dimensional Nanostructures towards Applications in Gas Sensing and CO Oxidation. *RSC Adv.* **2015**, *5*, 98500–98507, doi:10.1039/C5RA23446H.
35	Lou, Z.; Wang, L.; Wang, R.; Fei, T.; Zhang, T. Synthesis and Ethanol Sensing Properties of SnO2 Nanosheets via a Simple Hydrothermal Route. *Solid-State Electron.* **2012**, *76*, 91–94, doi:10.1016/j.sse.2012.05.062.
36	Naghadeh, S.B.; Vahdatifar, S.; Mortazavi, Y.; Khodadadi, A.A.; Abbasi, A. Functionalized MWCNTs Effects on Dramatic Enhancement of MWCNTs/SnO2

	Nanocomposite Gas Sensing Properties at Low Temperatures. *Sens. Actuators B Chem.* **2016**, *223*, 252–260, doi:10.1016/j.snb.2015.09.088.
37	Pan, Z.; Sun, F.; Xu, S.; Long, J.; Chen, Y.; Zhuang, Z. A General Electrodeposition-Based Method for in Situ Construction of Resistive-Type Semiconductor Film Gas-Sensor with Excellent Sensing Performance. *RSC Adv.* **2015**, *5*, 74075–74083, doi:10.1039/C5RA14153B.
38	Qiang, Z.; Ma, S.Y.; Jiao, H.Y.; Wang, T.T.; Jiang, X.H.; Jin, W.X.; Yang, H.M.; Chen, H. Highly Sensitive and Selective Ethanol Sensors Using Porous SnO2 Hollow Spheres. *Ceram. Int.* **2016**, *42*, 18983–18990, doi:10.1016/j.ceramint.2016.09.053.
39	Qin, G.; Gao, F.; Jiang, Q.; Li, Y.; Liu, Y.; Luo, L.; Zhao, K.; Zhao, H. Well-Aligned Nd-Doped SnO2 Nanorod Layered Arrays: Preparation, Characterization and Enhanced Alcohol-Gas Sensing Performance. *Phys. Chem. Chem. Phys.* **2016**, *18*, 5537–5549, doi:10.1039/C5CP07174G.
40	Qin, S.; Tang, P.; Feng, Y.; Li, D. Novel Ultrathin Mesoporous ZnO-SnO2 n-n Heterojunction Nanosheets with High Sensitivity to Ethanol. *Sens. Actuators B Chem.* **2020**, *309*, 127801, doi:10.1016/j.snb.2020.127801.
41	Qu, Y.; Wang, H.; Chen, H.; Han, M.; Lin, Z. Synthesis, Characterization and Sensing Properties of Mesoporous C/SnO2 Nanocomposite. *Sens. Actuators B Chem.* **2016**, *228*, 595–604, doi:10.1016/j.snb.2016.01.077.
42	Sankar, C.; Ponnuswamy, V.; Manickam, M.; Suresh, R.; Mariappan, R.; Vinod, P.S. Structural, Morphological, Optical and Gas Sensing Properties of Pure and Ce Doped SnO2 Thin Films Prepared by Jet Nebulizer Spray Pyrolysis (JNSP) Technique. *J. Mater. Sci. Mater. Electron.* **2017**, *28*, 4577–4585, doi:10.1007/s10854-016-6094-9.
43	Sun, G.-J.; Lee, J.K.; Lee, W.I.; Dwivedi, R.P.; Lee, C.; Ko, T. Ethanol Sensing Properties and Dominant Sensing Mechanism of NiO-Decorated SnO2 Nanorod Sensors. *Electron. Mater. Lett.* **2017**, *13*, 260–269, doi:10.1007/s13391-017-1719-6.
44	Tan, W.; Yu, Q.; Ruan, X.; Huang, X. Design of SnO2-Based Highly Sensitive Ethanol Gas Sensor Based on Quasi Molecular-Cluster Imprinting Mechanism. *Sens. Actuators B Chem.* **2015**, *212*, 47–54, doi:10.1016/j.snb.2015.01.035.
45	Tricoli, A.; Pratsinis, S.E. Dispersed Nanoelectrode Devices. *Nat. Nanotechnol.* **2010**, *5*, 54–60, doi:10.1038/nnano.2009.349.
46	Van Hieu, N.; Kim, H.-R.; Ju, B.-K.; Lee, J.-H. Enhanced Performance of SnO2 Nanowires Ethanol Sensor by Functionalizing with La2O3. *Sens. Actuators B Chem.* **2008**, *133*, 228–234, doi:10.1016/j.snb.2008.02.018.
47	Wang, B.; Sun, L.; Wang, Y. Template-Free Synthesis of Nanosheets-Assembled SnO2 Hollow Spheres for Enhanced Ethanol Gas Sensing. *Mater. Lett.* **2018**, *218*, 290–294, doi:10.1016/j.matlet.2018.02.003.
48	Wang, B.; Wang, Y.; Lei, Y.; Xie, S.; Wu, N.; Gou, Y.; Han, C.; Shi, Q.; Fang, D. Vertical SnO2 Nanosheet@SiC Nanofibers with Hierarchical Architecture for High-Performance Gas Sensors. *J. Mater. Chem. C* **2016**, *4*, 295–304, doi:10.1039/C5TC02792F.
49	Wang, C.; Cai, D.; Liu, B.; Li, H.; Wang, D.; Liu, Y.; Wang, L.; Wang, Y.; Li, Q.; Wang, T. Ethanol-Sensing Performance of Tin Dioxide Octahedral Nanocrystals with

	Exposed High-Energy {111} and {332} Facets. *J. Mater. Chem. A* **2014**, *2*, 10623–10628, doi:10.1039/C4TA00844H.
50	Wang, Q.; Yao, N.; An, D.; Li, Y.; Zou, Y.; Lian, X.; Tong, X. Enhanced Gas Sensing Properties of Hierarchical SnO2 Nanoflower Assembled from Nanorods via a One-Pot Template-Free Hydrothermal Method. *Ceram. Int.* **2016**, *42*, 15889–15896, doi:10.1016/j.ceramint.2016.07.062.
51	Wang, S.; Yu, W.; Cheng, C.; Zhang, T.; Ge, M.; Sun, Y.; Dai, N. Fabrication of Mesoporous SnO2 Nanocubes with Superior Ethanol Gas Sensing Property. *Mater. Res. Bull.* **2017**, *89*, 267–272, doi:10.1016/j.materresbull.2017.02.010.
52	Wang, T.T.; Ma, S.Y.; Cheng, L.; Luo, J.; Jiang, X.H.; Jin, W.X. Preparation of Yb-Doped SnO2 Hollow Nanofibers with an Enhanced Ethanol–Gas Sensing Performance by Electrospinning. *Sens. Actuators B Chem.* **2015**, *216*, 212–220, doi:10.1016/j.snb.2015.04.040.
53	Wang, Y.; Liu, C.; Wang, L.; Liu, J.; Zhang, B.; Gao, Y.; Sun, P.; Sun, Y.; Zhang, T.; Lu, G. Horseshoe-Shaped SnO2 with Annulus-like Mesoporous for Ethanol Gas Sensing Application. *Sens. Actuators B Chem.* **2017**, *240*, 1321–1329, doi:10.1016/j.snb.2016.07.160.
54	Xiao, L.; Shu, S.; Liu, S. A Facile Synthesis of Pd-Doped SnO2 Hollow Microcubes with Enhanced Sensing Performance. *Sens. Actuators B Chem.* **2015**, *221*, 120–126, doi:10.1016/j.snb.2015.06.099.
55	Xie, N.; Guo, L.; Chen, F.; Kou, X.; Wang, C.; Ma, J.; Sun, Y.; Liu, F.; Liang, X.; Gao, Y.; et al. Enhanced Sensing Properties of SnO2 Nanofibers with a Novel Structure by Carbonization. *Sens. Actuators B Chem.* **2018**, *271*, 44–53, doi:10.1016/j.snb.2018.05.039.
56	Xu, M.-H.; Cai, F.-S.; Yin, J.; Yuan, Z.-H.; Bie, L.-J. Facile Synthesis of Highly Ethanol-Sensitive SnO2 Nanosheets Using Homogeneous Precipitation Method. *Sens. Actuators B Chem.* **2010**, *145*, 875–878, doi:10.1016/j.snb.2010.01.002.
57	Xu, W.; Xia, L.; Ju, J.; Xi, P.; Cheng, B.; Liang, Y. Preparation and Low-Temperature Gas-Sensing Properties of SnO2 Ultra-Fine Fibers Fabricated by a Centrifugal Spinning Process. *J. Sol-Gel Sci. Technol.* **2016**, *78*, 353–364, doi:10.1007/s10971-016-3962-y.
58	Xue, N.; Zhang, Q.; Zhang, S.; Zong, P.; Yang, F. Highly Sensitive and Selective Hydrogen Gas Sensor Using the Mesoporous SnO2 Modified Layers. *Sensors* **2017**, *17*, 2351, doi:10.3390/s17102351.
59	Yan, S.; Liang, X.; Song, H.; Ma, S.; Lu, Y. Synthesis of Porous CeO2-SnO2 Nanosheets Gas Sensors with Enhanced Sensitivity. *Ceram. Int.* **2018**, *44*, 358–363, doi:10.1016/j.ceramint.2017.09.181.
60	Yang, F.; Guo, Z. Comparison of the Enhanced Gas Sensing Properties of Tin Dioxide Samples Doped with Different Catalytic Transition Elements. *J. Colloid Interface Sci.* **2015**, *448*, 265–274, doi:10.1016/j.jcis.2015.02.045.
61	Yoon, J.-W.; Choi, S.H.; Kim, J.-S.; Jang, H.W.; Kang, Y.C.; Lee, J.-H. Trimodally Porous SnO2 Nanospheres with Three-Dimensional Interconnectivity and Size Tunability: A One-Pot Synthetic Route and Potential Application as an Extremely Sensitive Ethanol Detector. *NPG Asia Mater.* **2016**, *8*, e244, doi:10.1038/am.2016.16.

62	Yu, X.; Zeng, W. Fabrication and Gas-Sensing Performance of Nanorod-Assembled SnO2 Nanostructures. *J. Mater. Sci. Mater. Electron.* **2016**, *27*, 7448–7453, doi:10.1007/s10854-016-4721-0.
63	Yue, L.; Ge, J.; Luo, G.; Bian, K.; Yin, C.; Guan, R.; Zhang, W.; Zhou, Z.; Wang, K.; Guo, X. A Facile Large-Scale Synthesis of Porous SnO2 by Bronze for Superior Lithium Storage and Gas Sensing Properties Through a Wet Chemical Reaction Strategy. *J. Electron. Mater.* **2018**, *47*, 2545–2556, doi:10.1007/s11664-018-6130-z.
64	Zeng, Q.; Cui, Y.; Zhu, L.; Yao, Y. Increasing Oxygen Vacancies at Room Temperature in SnO2 for Enhancing Ethanol Gas Sensing. *Mater. Sci. Semicond. Process.* **2020**, *111*, 104962, doi:10.1016/j.mssp.2020.104962.
65	Zeng, W.; He, Q.; Pan, K.; Wang, Y. Synthesis of Multifarious Hierarchical Flower-like SnO2 and Their Gas-Sensing Properties. *Phys. E Low-Dimens. Syst. Nanostructures* **2013**, *54*, 313–318, doi:10.1016/j.physe.2013.07.014.
66	Zeng, W.; Li, T.; Li, T.; Hao, J.; Li, Y. Template-Free Synthesis of Highly Ethanol-Response Hollow SnO2 Spheres Using Hydrothermal Process. *J. Mater. Sci. Mater. Electron.* **2015**, *26*, 1192–1197, doi:10.1007/s10854-014-2524-8.
67	Zeng, W.; Zhang, H.; Li, Y.; Chen, W.; Wang, Z. Hydrothermal Synthesis of Hierarchical Flower-like SnO2 Nanostructures with Enhanced Ethanol Gas Sensing Properties. *Mater. Res. Bull.* **2014**, *57*, 91–96, doi:10.1016/j.materresbull.2014.05.019.
68	Zhang, B.; Fu, W.; Meng, X.; Ruan, A.; Su, P.; Yang, H. Enhanced Ethanol Sensing Properties Based on Spherical-Coral-like SnO2 Nanorods Decorated with α-Fe2O3 Nanocrystallites. *Sens. Actuators B Chem.* **2018**, *261*, 505–514, doi:10.1016/j.snb.2018.01.133.
69	Zhang, L.; Tong, R.; Ge, W.; Guo, R.; Shirsath, S.E.; Zhu, J. Facile One-Step Hydrothermal Synthesis of SnO2 Microspheres with Oxygen Vacancies for Superior Ethanol Sensor. *J. Alloys Compd.* **2020**, *814*, 152266, doi:10.1016/j.jallcom.2019.152266.
70	Zhang, R.; Jia, J.-B.; Cao, J.-L.; Wang, Y. SnO2/Graphene Nanoplatelet Nanocomposites: Solid-State Method Synthesis With High Ethanol Gas-Sensing Performance. *Front. Chem.* **2018**, *6*, 337, doi:10.3389/fchem.2018.00337.
71	Zhang, Y.; He, X.; Li, J.; Miao, Z.; Huang, F. Fabrication and Ethanol-Sensing Properties of Micro Gas Sensor Based on Electrospun SnO2 Nanofibers. *Sens. Actuators B Chem.* **2008**, *132*, 67–73, doi:10.1016/j.snb.2008.01.006.
72	Zhao, R.; Wang, Z.; Zou, T.; Wang, Z.; Xing, X.; Yang, Y.; Wang, Y. 'Green' Prepare SnO2 Nanofibers by Shaddock Peels: Application for Detection of Volatile Organic Compound Gases. *J. Mater. Sci. Mater. Electron.* **2019**, *30*, 3032–3044, doi:10.1007/s10854-018-00582-5.
73	Zhao, T.; Qiu, P.; Fan, Y.; Yang, J.; Jiang, W.; Wang, L.; Deng, Y.; Luo, W. Hierarchical Branched Mesoporous TiO2–SnO2 Nanocomposites with Well-Defined n–n Heterojunctions for Highly Efficient Ethanol Sensing. *Adv. Sci.* **2019**, *6*, 1902008, doi:10.1002/advs.201902008.
74	Zhou, Q.; Chen, W.; Li, J.; Tang, C.; Zhang, H. Nanosheet-Assembled Flower-like SnO2 Hierarchical Structures with Enhanced Gas-Sensing Performance. *Mater. Lett.* **2015**, *161*, 499–502, doi:10.1016/j.matlet.2015.09.010.

75	Zito, C.A.; Perfecto, T.M.; Volanti, D.P. Impact of Reduced Graphene Oxide on the Ethanol Sensing Performance of Hollow SnO2 Nanoparticles under Humid Atmosphere. *Sens. Actuators B Chem.* **2017**, *244*, 466–474, doi:10.1016/j.snb.2017.01.015.
76	Zito, C.A.; Perfecto, T.M.; Volanti, D.P. Palladium-Loaded Hierarchical Flower-like Tin Dioxide Structure as Chemosensor Exhibiting High Ethanol Response in Humid Conditions. *Adv. Mater. Interfaces* **2017**, *4*, 1700847, doi:10.1002/admi.201700847.
